# Grey-Wolf-Based Wang’s Demons for Retinal Image Registration

**DOI:** 10.3390/e22060659

**Published:** 2020-06-15

**Authors:** Sayan Chakraborty, Ratika Pradhan, Amira S. Ashour, Luminita Moraru, Nilanjan Dey

**Affiliations:** 1 Department of Computer Applications, SMIT, Sikkim Manipal University, Sikkim 737136, India; sayan.cb@gmail.com (S.C.); ratika.p@smit.smu.edu.in (R.P.); 2 Department of Electronics and Electrical Communications Engineering, Faculty of Engineering, Tanta University, Tanta 31527, Egypt; amirasashour@yahoo.com or; 3 Department of Chemistry, Physics & Environment, Faculty of Sciences and Environment, Dunarea de Jos University of Galati, 47 Domneasca Str., 800008 Galati, Romania; 4 Department of Information Technology, Techno International New Town, West Bengal 700156, India

**Keywords:** demons registration, firefly algorithm, cuckoo search, grey-wolf optimization, correlation, image registration

## Abstract

Image registration has an imperative role in medical imaging. In this work, a grey-wolf optimizer (GWO)-based non-rigid demons registration is proposed to support the retinal image registration process. A comparative study of the proposed GWO-based demons registration framework with cuckoo search, firefly algorithm, and particle swarm optimization-based demons registration is conducted. In addition, a comparative analysis of different demons registration methods, such as Wang’s demons, Tang’s demons, and Thirion’s demons which are optimized using the proposed GWO is carried out. The results established the superiority of the GWO-based framework which achieved 0.9977 correlation, and fast processing compared to the use of the other optimization algorithms. Moreover, GWO-based Wang’s demons performed better accuracy compared to the Tang’s demons and Thirion’s demons framework. It also achieved the best less registration error of 8.36 × 10^−5^.

## 1. Introduction

Retinal image registration [1,2] has an essential role to assist the ophthalmologist in the diagnosis of eye diseases, evaluate the disease and their growth rate, screen the patient’s screening, and to assist laser surgical procedures. The blood vessels in the retina [3] can be directly imaged noninvasively, such as the fundus images and fluorescein angiogram (FA) images [4,5]. The most common image registration methods include area-based, feature-based, and hybrid registration methods. Area-based registration is dependent mainly on the intensity of pixels. There are some optimized functions as well which play an integral part in this case, such as mean square error, mutual information [6], similarity measurement [7], correlation (cross and phase) [8], etc. Statically, a window of points is compared among the input and reference images [9]. Two key points affect the performance of such a system, they are large homogeneous [10] and non-uniform. In the feature-based registration methods, the features (common structures) are usually extracted and matched from reference and sensed images, where the point correspondences are available in both images. The registration process is performed by maximizing a similarity measure computed from the correspondences [10]. The performance of feature-based methods depends on the sufficiency and reliability of the correspondences. These methods consist of various steps, including feature detection, feature matching, transform model estimation, and image resampling and transformation [11]. Fovea, optic disk [12,13], and retinal blood vessels [14] are some of the key features found in fundus retinal images [15]. A large number of existing works, used these features. Fundus’ images brightest component [16] is the optic disk (OD). Hence, it is easier to detect pixels with a higher intensity as they have higher grayscale value. On the contrary, the fovea is the least bright part in the retinal image. A priori knowledge of the optic disk position helps to understand the gap between the fovea and optic disk. The gap [13] between the center of OD and fovea is 2.5 times the diameter of the optic disk. Vessel tracking [17], matched filters [18], morphological processing [19], pixel-based classifiers such as neural networks helps to obtain or extract the retinal blood vessels from retinal images. The hybrid image registration algorithm includes both area and feature-based registration methods [10,11,12,13,14,15,16,17,18,19,20,21].

One of the key objectives of image registration is to get rid of image registration error and make it more efficient than the existing techniques [22] with avoiding the time complexity [1,2,3,4]. Image registration refers to aligning one or more images [23,24] with reference to other images [5]. It depends on the concept of image transformation [6], in which an image or image matrix uses various operations, such as translation, rotation, scaling, shearing, [25] and mapping. Based on such operations, image registration [26,27,28] can be divided into two different categories: rigid and non-rigid [29]. Rigid registration [30] consists of rotation, translation, and scaling. Unlike the rigid registration which may involve various geometric transformations [31] without changing the shape of the object, the non-rigid registration [32,33] is based on the same concept but affects the shape of the objects involved in the image registration process [1]. Non-rigid registration can be applied to mono-modal as well as multimodal images, where the transformation can include the change of the object’s shapes to warp [34] the retinal images [19]. For retinal image registration, non-rigid registration [35] involves 3D eye motion, and image acquisition parameters which are imprecisely known for retinal image databases. Hence it is essential to register the retinal images to find out the correct pairs, obtain the ground-truth, and identify the difference in images by overlapping them with respect to one another.

Demons registration is an efficient non-rigid image registration technique due to its fluid registration mechanism, which allows it to have much lesser distortion of objects compared to other non-rigid registrations [7]. It changes the shape of the object [8,9]. Since the registration process includes various parameters, the optimization of image registration becomes essential in different registration processes to determine the optimal parameters for superior results [16,17,18]. Different optimization algorithms proved their efficiency in the different medical imaging applications, including cuckoo search (CS), firefly algorithm (FA), particle swarm optimization (PSO), and grey-wolf optimization (GWO) algorithm [36,37]. In addition, the GWO algorithm [10,11] is considered one of the efficient optimization [38] methods for medical image registration and fusion. For instance, the GWO was applied to identify scale selection from brain images to improve image fusion technique [12] as well as to enhance [39] the homomorphic wavelet-based fusion [13].

In the current work, the grey-wolf optimization (GWO) algorithm [40,41] is applied to build up the optimization framework to achieve an optimal solution [42] for better accuracy and faster image non-rigid demons registration [11,22,23,24,25,26]. The current work focuses on reducing the registration error as well as the time complexity in order to get rid of the problem raised by processing on large chunk of images in retinal image database, that takes a large amount of time. A comparative study is conducted with the cuckoo search- [10], firefly algorithm [43], and particle swarm optimization- [9] based demons registration [44,45,46] framework.

The following sections of the current work include the literature survey in Section 2 followed by introducing the various types of demons algorithm-based image registration in Section 3. Section 4 discusses the optimization frameworks. The proposed method is explained in Section 5, where the results are observed and analyzed in Section 6 and discussed in Section 7. The conclusion of the present work is highlighted in Section 8.

## 2. Literature Survey

Retinal image registration is an essential tool in medical imaging which attracts several researchers to develop new methods. In 2012, Gharabaghi et al. [3] proposed a retinal image registration method using geometrical features by applying for non-rigid affine registration on retinal images obtained from the DRIVE database. Ramli et al. [1] proposed a feature-based retinal image registration using a D-Saddle-based detector to identify the feature points in retinal images obtained from the FIRE dataset. Hernandez-Matas et al. [2] designed a retinal image registration method based on the concept of the spherical shape of the human eye, where each image was warped with respect to another image using rigid registration. Later in 2017, Zhuang et al. [47] proposed a deformable image registration algorithm based on improved active demons using the length and area of connected region as a driving force to build an active demons diffusion equation. In 2018, Li et al. [4] implemented a multimodal and multivendor retinal image registration using deformable registration based on modality independent neighborhood descriptor (MIND) framework on color fundus images. To improve the demons registration of medical images, Mishra et al. [46] used the 2D image registration method on mono-modal images. To obtain the deformation fields of medical images, the velocity field of demons was evaluated. In 2016, Lan et al. [43] proposed a comparative analysis of all demons registration, which proved that Wang’s demons [43] was superior in terms of performance than Thirion’s and Tang’s demons.

On the other hand, due to the effectiveness of the optimization algorithms [48] in the registration [49] process, several researchers employed several optimizers to develop the registration process. Zhang and Wu [50] applied a firefly algorithm-based rigid image registration to improve the traditional rigid registration method compared to the use of particle swarm optimization and genetic algorithm as benchmarks. The firefly algorithm was applied to spatial transformation parameters and was compared with genetic algorithm (GA), PSO, and Artificial bee Colony (ABC) algorithm. The results showed that the firefly algorithm performed better than the ABC, PSO, and GA, respectively, where the computation time of FA was only 1.0683s, slower than the ABC, but yet faster than GA and PSO. Cocianu and Stan [39] developed an image registration technique based on the firefly algorithm to realize less registration error and time complexity reduction. Ayatollahi et al. [33] proposed a PSO-based multimodal brain image registration using mutual information of the original and resultant image as the fitness function. The translation, rotation, and scaling were used as the three parameters to be optimized using hybrid particle swarm optimization. The root mean square error was measured between the original and registered images as the fitness function. The PSO-based framework had less error 0.496 compared to the 0.888 error when using the GA-based framework. Zhang et al. [50] discussed the effect of the FA on rigid registration by optimizing the rotational parameter of rigid registration to achieve the lowest error. Xiaogang et al. [51] applied the FA firefly algorithm for efficient image registration using mutual information as the fitness function. Moreover, Chakraborty et al. [44] used the firefly algorithm-based image registration framework that involved the optimization of Thirion’s demons. Afterward, Chakraborty et al. [21] introduced a CS algorithm-based image registration compared to the FA-based demons registration. In 2018, Yogapriya et al. [48] introduced a fuzzy-based grey-wolf optimization technique for the medical image retrieval system using image texture features. This system classified the images based on the features and reduced the high dimensional texture features using fuzzy-based grey-wolf optimization. Recently in 2019, Asha et al. [49] presented a multi-modal medical image fusion technique combined with grey-wolf optimization.

From the preceding reported studies, it is obvious that the medical image registration techniques lack a proper direction of optimization [52,53] of the problem of registration error [54,55] and time complexity [56]. Hence, the current work aims to address such problems during image registration.

## 3. Non-Rigid Image Registration Using Demons Algorithm

Image registration is based on the concept of image transformation of computer graphics. The basic transformations involved in registration are translation, rotation, and scaling. In this transformation, the object’s shape does not change, only their viewing perspective changes from frame to frame. Hence, these types of transformations or registrations were called rigid registrations. To make the animations more realistic, a dynamic change of the frame and objects are required. Hence, mapping, shearing, and deformation become significant in the transformation. These operations not only change the object’s shape, it also deformed other parts of the frames. However, the animations looked more realistic as deformation and restoration of shapes in continuous frames made the visual livelier than rigid registration. These transformations were known as non-rigid transformations. Initially, shearing and mapping were included with the rigid transformation to introduce affine registration. Later, spline curves came into play to modify the mapping of objects from frame to frame. Neural [57,58] or brain images [59,60] have been a key area to apply such non-rigid registration. Nevertheless, all these non-rigid registrations majorly lacked the concept of changing the shape of the object in such a way that it becomes imperceptible by the human eye. Hence, demons registration was originated by Thirion [30], and modified by Wang et al. [61] and Tang et al. [28]. It is known as fluid registration for its superior performance over other non-rigid registrations. For image matrix transformation, it operates through displacement deviator among the involved images. Pixel velocity and edge-based forces help to obtain the displacement deviator. These are the main three demons registrations that has been widely used on medical images as follows.

### 3.1. Thirion’s Demons

The concept of the demons registration by Thirions is based on the optical flow, where the demons force refers to the displacement vector applied by the reference image (R) on the deformed image. The pixels in the registered/deformed image are shifted to get aligned based on the reference image. Since the demons registration process is iterative, the deformation field is the outcome of after each iteration. The objective function should have the smoothness of the alignment field as well as similarity measurement, which is defined by [30]:

(1) $$E(\vec{u})=||R-F⊗\vec{u}|{|}^{2}+\sigma^{2}||\vec{u}|{|}^{2}$$

where the reference and query images are denoted by *R*, and *F*, respectively. Additionally, the deformation field $\vec{u}$ is updated after each iteration, where the deformation operation is mentioned as ⊗. The Gaussian smoothening kernel σ is applied in demons, which is a variable parameter. To calculate the value of $\vec{u}$, the following formula is applied [30,43]:

(2) $$\vec{u}=(||R-F||)(\frac{\vec{\nabla R}}{||\vec{\nabla R}|{|}^{2}+||R-F|{|}^{2}})$$

where $\vec{\nabla R}$ is the gradient information of the reference image. The gradient information are calculated using

(3) $$\nabla F=\frac{\partial F}{\partial x}\hat{i}+\frac{\partial F}{\partial y}\hat{j}$$

(4) $$\nabla R=\frac{\partial R}{\partial x}\hat{i}+\frac{\partial R}{\partial y}\hat{j}$$

The default values of the registration parameters as defined by Thirion are listed in Table 1.

### 3.2. Wang’s Demons

In Wang’s demons, the registration approach was changed slightly, where Wang et al. [61] modified Thirion’s demons by introducing gradient information $\vec{\nabla R}$ of the reference image *R*. The problem in Thirion’s demons provides the deformation field information with respect to the reference image *R*. Typically, Thirion’s demons is effective for small deformed problems as it collects information from deformation field. Hence, Wang’s demons [2,19,61] introduces the gradient information managed to eradicate these problems. The equation of Wang’s demons force is given by:

(5) $$\vec{u}=(||R-F||)(\frac{\vec{\nabla R}}{||\vec{\nabla R}|{|}^{2}+\alpha^{2}||R-F|{|}^{2}}+\frac{\vec{\nabla F}}{||\vec{\nabla F}|{|}^{2}+\alpha^{2}||R-F|{|}^{2}})$$

where α is the noise constant which drive the force strength to be stabilized in every iteration. The alignment field is calculated by applying the Gaussian smoothening kernel in demons is *σ* [30] using the following formula:

(6) $$E(\vec{u})=||R-F⊗\vec{u}|{|}^{2}+\sigma^{2}||\vec{u}|{|}^{2}$$

In the present work, the value of *σ* is optimized.

### 3.3. Tang’s Demons

Since the analysis of the general range of parameters was still missing in demons registration, Tang et al. [4] introduced a balance coefficient *k* in Tang’s demons to solve this problem. The adaptation of the balance coefficient is managed to adjust the demons force and its strength, where the Tang’s demons force is represented by [28,43]:

(7) $$\vec{u}=(||R-F||)(\frac{\vec{\nabla R}}{k^{2}||\vec{\nabla R}|{|}^{2}+\alpha^{2}||R-F|{|}^{2}}+\frac{\vec{\nabla F}}{k^{2}||\vec{\nabla F}|{|}^{2}+\alpha^{2}||R-F|{|}^{2}})$$

where $\vec{e\nabla R}$ and $\vec{e\nabla F}$ are the gradient field information unit vectors of the reference image (*R*), and fixed image (*F*), respectively. The Tang’s demons force is given by:

(8) $$\vec{u}=(||R-F||)(\frac{\vec{\nabla R}}{k^{2}||\vec{\nabla R}|{|}^{2}+\alpha^{2}||R-F|{|}^{2}}\vec{e\nabla R}+\frac{\vec{\nabla F}}{k^{2}||\vec{\nabla F}|{|}^{2}+\alpha^{2}||R-F|{|}^{2}}\vec{e\nabla F})$$

Thus, despite the balance coefficient *k,* Wang’s demons proved its efficiency compared to the Tang’s demons [43]. The unit vectors of the gradient field are managed to simplify the Thirion’s demons leading to producing worse results than Wang’s demons. It has been observed that without these parameters, demons registration can produce better results.

## 4. Optimization Framework for Image Registration

A grey-wolf optimization algorithm is applied in the present work to build the framework for demons registration. The main principle of the grey-wolf optimizer (GWO) is originated from the behaviors and characteristics of grey wolf [41]. The grey-wolf optimizer is one of the most advanced meta-heuristic algorithms. It is based on the adaptation of the grey-wolf’s hunting mechanism. The algorithm divides the group of grey wolves into four different subgroups: Alpha (α), Beta (β), Delta (δ), and Omega (ω). The hunting area is considered as the search area of the problem statement, where alpha-, beta-, and delta- wolves direct the omega wolves to the favorable zone. During the iterative process, alpha, beta, and delta change their position based on the following expression [40,41]:

(9) $$D=|\vec{W}p(t).\vec{C}-\vec{W}(t)|$$

(10) $$\vec{W}(t+1)=\vec{W}p(t)-\vec{D}\cdot \vec{A}$$

where $\vec{D}$ represents the gap between the prey and predator’s position, *t* is the current iteration and $\vec{W}p(t)$ is the prey’s position. The grey-wolf’s position is denoted by $\vec{W}(t)$, which is updated in Equation (8), where *A* and *C* are determined using the following expressions [40,41]:

(11) $$\begin{array}{l} \vec{A}={\vec{r}}_{1}a\cdot 2a\\ \vec{C}={\vec{r}}_{2}\cdot 2 \end{array}$$

where ${\vec{r}}_{1}$ and ${\vec{r}}_{2}$ are two vectors having random values between 0 and 1, while the value of *a* varies between 0 to 2 and is gradually decreased from 2 to 0. Hence, the first three best solutions [40,41] values (Alpha (α), Beta (β), Delta (δ)) are initialized and updated. These values assist other search agents, including Omegas (ω) to update their positions according to the position of the best search agents. Accordingly, the following formulas help to calculate and update the position of grey wolves [40,41]:

(12) $${\vec{D}}_{\alpha }=\left|C_{1}\cdot {\vec{W}}_{\alpha }-\vec{W}\right|,{\vec{D}}_{\beta }=\left|C_{2}\cdot {\vec{W}}_{\beta }-\vec{W}\right|,{\vec{D}}_{\delta }=\left|C_{3}\cdot {\vec{W}}_{\delta }-\vec{W}\right|$$

(13) $${\vec{W}}_{1}={\vec{W}}_{\alpha }-{\vec{A}}_{1}.\left({\vec{D}}_{\alpha }\right),{\vec{W}}_{2}={\vec{W}}_{\beta }-{\vec{A}}_{2}.\left({\vec{D}}_{\beta }\right),{\vec{W}}_{3}={\vec{W}}_{\delta }-{\vec{A}}_{3}.\left({\vec{D}}_{\delta }\right)$$

The Algorithm 1 of the GWO is reported as follows.
**Algorithm 1 Grey-Wolf Optimizer (GWO) procedure**  ***Start***    ***Initialize*** the population *W*_i_ (*i* = 1,2,..., *n*) and *a*, *A* and *C* in GWO    ***Evaluate***
*the* search agent’s fitness value      *W_α_* denotes the best search agent      *W_β_* denotes next best search agent      *W_δ_* denotes 3^rd^ best search agent    ***While***(*t* < Maxgeneration)       ***For*** each search agent          ***Update*** search agent’s current position       ***End for***       ***Update*** values of *a*, *A* and *C*    ***Evaluate** search agents’ fitness values*    ***Update***
*W_α_, W_β,_ and W_δ_*    *t++*    ***End while***     ***Return*** W_α_   ***Stop***

In this work, the fitness function is the correlation coefficient which is used as the objective function. The correlation coefficient is a good metric of understanding the amount of difference between two images to determine the amount of similarity between the original and registered image [27]. The main aim of the proposed work is to increase the registration accuracy, i.e., increase the image quality [29,43], so that the registered image is almost identical to the target image. Henceforth, the correlation coefficient was chosen as the objective function in this current work. Accordingly, the proposed algorithm-based Wang’s demons registration is given in Algorithm 2.
**Algorithm 2: Proposed algorithm based Wang’s Demons Registration*****Start***  ***Read*** the source and target images  ***Set*** upper bound and lower bound for optimization  ***Apply*** the meta-heuristic algorithm (GWO) for optimization  ***Generate*** new solutions  ***For*** solution 1 to *n*       ***Apply*** the solutions on demons algorithm            ***Perform*** demons algorithm for each solution and return the correlation value between the original and registered images       ***Choose*** the highest correlation value and return it as best fit       ***Obtain*** the solution values for which best fit was returned       ***Save*** the registered image for post-processing  ***End for***       ***Calculate*** the mean square error, joint entropy, and mutual information following the optimization of demons registration***Stop***

## 5. Proposed Method

**Figure 1:**
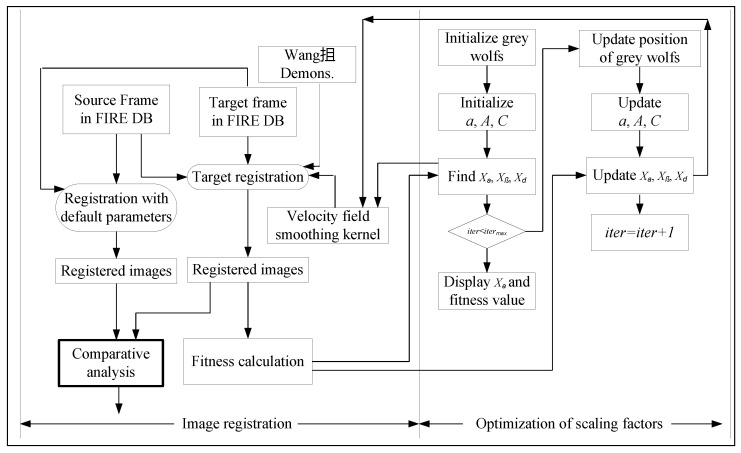
Block diagram of the proposed method.

As previously discussed in Section 3, the objective function of non-rigid registration [42,43] must include the alignment field’s similarity measurement. The present optimizes the parameters of Wang’s demons [61], where it is faster and has less error in demons deformation during the registration process compared to the Wang’s demons [61], and the Thirion’s demons [30]. Hence, Wang’s demons is applied in this current work for the image registration framework to determine the optimal value of the alpha noise constant and Gaussian smoothening filter. Typically, the default window size in Thirion’s [30] demons as well as Wang’s demons [61] is 60 × 60, and the sigma value is 10. The block diagram of the proposed method is illustrated in Figure 1.

In the proposed method, the retinal images [62,63] are converted initially into gray-scale images. The image frames [64] were then processed for demons registration. Correlation value [57,58,59] of the original and registered image is considered as the best fitness. The parameters of the demons registration are the scaling factors to be optimized using the GWO. The image with the highest correlation [60] with the original image is chosen to be the optimal registered image and the scaling factors value as well as the correlation value were stored for post-processing. Figure 1 explains the proposed work, where the left side the image registration part is carried out, in which, firstly the source and then target frames are chosen from the database. The registration is done using the default parameters, and optimized parameters that include the Gaussian filter’s window size (60 × 60 pixels), and 2-D sigma value (10). These values are varied from 20 to 100 values for window size and 0–20 for 2-D sigma value during optimization, using grey-wolf optimization. The grey-wolf optimizer can be observed on the right side of the diagram. It uses the correlation value between the original and registered images to find the best fitness and respective parameter values. After each iteration, values, or position of grey wolves get updated, i.e., the values of the parameters are chosen from the population pool, and the registration is done over and over again until the best fitness is found.

## 6. Results and Discussion

The current work is tested and executed on Intel i3, 2.2 GHz processor-based system with 8 GB ram, and Windows 10 operating system. The simulation for running the image registration process was done on MatLab R2018a software in which CS, PSO, FA, genetic algorithm (GA), and the proposed GWO has been applied for optimization of the registration framework. The 31st pair from P-set in the FIRE database is chosen for image registration [19].

### 6.1. Dataset

The dataset used in this current work was one of the popular datasets available for retinal image processing, named as FIRE-DB [65]. There are 129 retinal images inside the FIRE-DB forming a total of 134 image pairs. These image pairs are further classified and stored according to their different categories. There are three categories: set A, set P, and set S. These images were captured using Nidek AFC-210 fundus camera. The resolution of all images was 2912 × 2912 pixels as obtained by the camera. The FOV (Field of View) of images was set at 45° in x and y dimensions. The set of capturing the images was done in Papageorgiou Hospital, Aristotle University of Thessaloniki. A total of 39 patients volunteered for this task. Among these datasets, the same image pair was chosen for the current framework, as discussed in Lan et al. [43]

### 6.2. Meta-Heuristics Based Wang’s Demons Registration

Several performance evaluation metrics are measured, where the correlation between two images (X and Y, X’ and Y’ are mean of the images) is measured in terms of the correlation coefficient which is given by [39]:

(14) $$corr=\frac{\sum_{m}\sum_{n}(X_{\mathrm{mn}}-X^{\prime })(Y_{\mathrm{mn}}-Y^{\prime })}{\sqrt{\left(\sum_{m}\sum_{n}{\left(X_{\mathrm{mn}}-X^{\prime }\right)}^{2})(\sum_{m}\sum_{n}{\left(Y_{\mathrm{mn}}-Y^{\prime }\right)}^{2}\right)}}$$

In the present work, several optimization algorithms, including FA, CS, PSO, and GA, are applied to optimize Wang’s demons registration parameters for comparative study with the proposed GWO-based registration.

#### 6.2.1. Grey-Wolf Optimization-Based Wang’s Demons Registration

Table 2 reported the $k_{1},\;k_{2}$, and $k_{3}$ values for grey-wolf optimization algorithm-based demons registration framework. The population has been kept fixed at 15, while iterations are increased by 5, from the 5th generation to the 30th.

**Figure 2:**
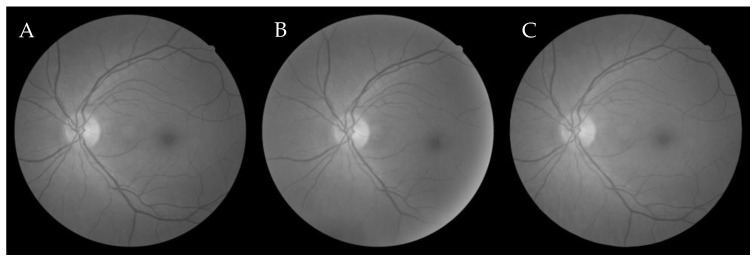
Moving (**A**), fixed (**B**), and registered (**C**) image in grey-wolf optimization-based demons registration.

The observation shows that it took 20 iterations to reach convergence and the best fitness value was 0.9977 which was the correlation value between the original and registered image that was achieved by the Gaussian filter value of 99 × 99 and sigma value of 20. The images involved and the resultant image is shown in Figure 2.

#### 6.2.2. Firefly Algorithm-Based Wang’s Demons Registration

**Figure 3:**
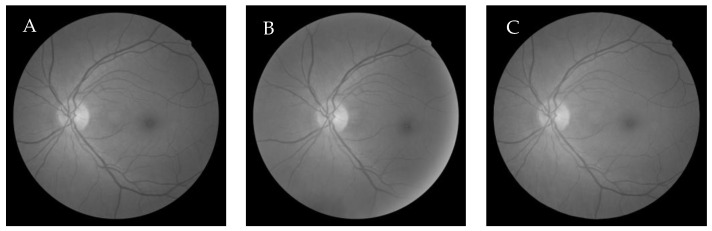
Moving, fixed, and registered image in firefly algorithm-based demons registration, where (**A**) reference retinal image, (**B**) original image, and (**C**) registered image using FA-based Wang’s demons registration.

The FA is used to optimize three parameters namely, namely $k_{1},\;k_{2}$, and $k_{3}$ for the demons registration framework. Figure 3 includes a sample of the moving, fixed, and registered image using the firefly algorithm-based demons registration.

In addition, Table 3 reported the evaluation metrics’ value of the used FA-based Wang’s demons registration, the required optimization time, and the optimal valued of the optimized parameters of Wang’s demons registration method.

The observation shows that it took 20 iterations for the FA to reach convergence and the best fitness value was 0.9970 that was the correlation value between original and registered image.

#### 6.2.3. Cuckoo search algorithm-based Wang’s Demons registration

Table 4 reported the $k_{1},\;k_{2}$, and $k_{3}$ values for the cuckoo search algorithm-based demons registration framework. The population has been kept fixed at 15, while iterations are increased by 5, from the 5th generation to the 30th.

**Figure 4:**
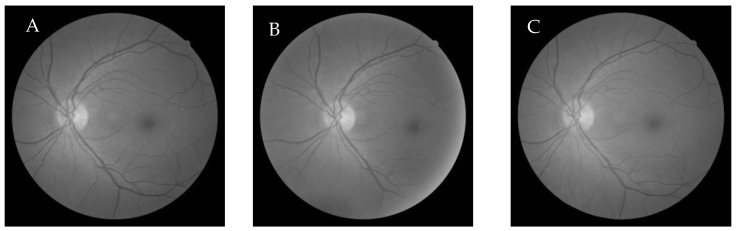
Moving (**A**), fixed (**B**), and registered (**C**) image in cuckoo search algorithm-based demons registration.

The observation shows that it took 20 iterations to reach convergence and the best fitness value was 0.9970 which was the correlation value between the original and registered image that was achieved by the Gaussian filter value of 99 × 99 and sigma value of 20. Clearly, the results are better than the previous two methods (FA and GWO). Figure 4 shows the images involved and the resultant image.

#### 6.2.4. Genetic Algorithm-Based Wang’s Demons Registration

The same observation was done using the Genetic algorithm as well and the results are shown in Table 5. Same as the previous three methods, here also the population has been kept fixed at 15, while iterations are increased by 5, from the 5th generation to the 30th.

**Figure 5:**
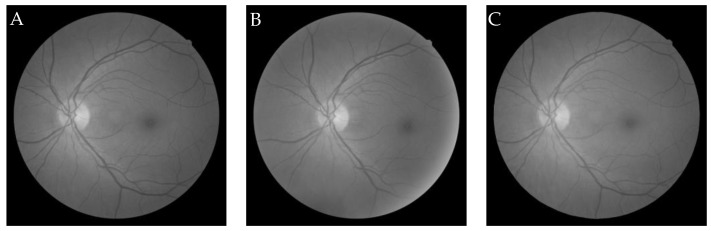
Moving (**A**), fixed (**B**), and registered (**C**) image in genetic algorithm-based demons registration.

The original image, registered image, and reference image involved in the genetic algorithm-based framework for Wang’s demons registration are illustrated below in Figure 5.

#### 6.2.5. Particle Swarm Optimization Algorithm-Based Wang’s Demons Registration

To keep the analysis simple, the same process was continued for particle swarm optimization as well and the results are shown in Table 6. Same as the previous three methods, here also the population has been kept fixed at 15, while iterations are increased by 5, from the 5th generation to the 30th.

**Figure 6:**
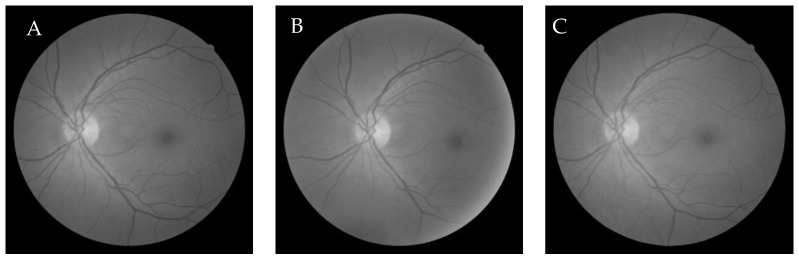
Moving (**A**), fixed (**B**), and registered (**C**) image in particle swarm optimization-based demons registration.

The results are poorer compared to the previous three methods, as the framework took longer time to optimize as well as, the resultant images are no better than the firefly algorithm or grey-wolf optimization-based algorithm results. The original images involved and the resultant image are shown in Figure 6.

As previously discussed, the fundus images that were used were taken from fire-DB, and a pair of fundus images were chosen from the database, that was used in Lan et al.’s work [43].

The values of the parameters that were optimized were the window size of the Gaussian low pass filter, with a range varying from 20–100 and sigma range varying from 0–20 as reported in Table 7.

#### 6.2.6. Comparative Study in Terms of the Time Complexity and the Fitness Values Using Wang’s Demons Registration

**Figure 7:**
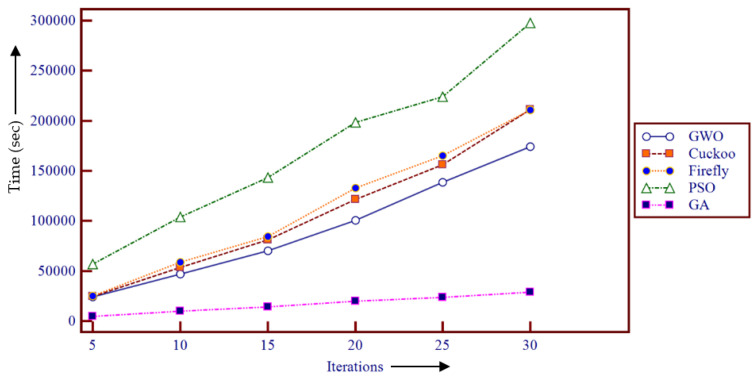
Comparative study of time complexity in meta-heuristic algorithm-based demons registration.

The time complexity of the used meta-heuristic framework-based demons registration is analyzed as illustrated in Figure 7.

**Figure 8:**
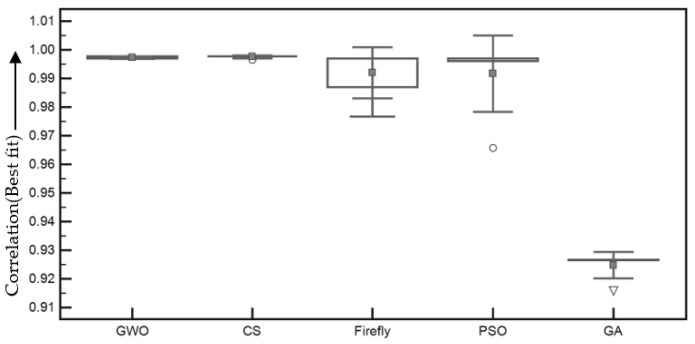
Comparative study of fitness values in meta-heuristic algorithm-based demons registration.

Figure 7 revealed that GA achieved the fastest convergence during the optimization process. However, the GWO performed superior results in terms of the time complexity compared to the FA-, GA-, and PSO-based registration methods. Moreover, Figure 8 demonstrates the box plot of the comparative study in terms of the achieved fitness values using the different meta-heuristic algorithm-based demons registration.

**Figure 9:**
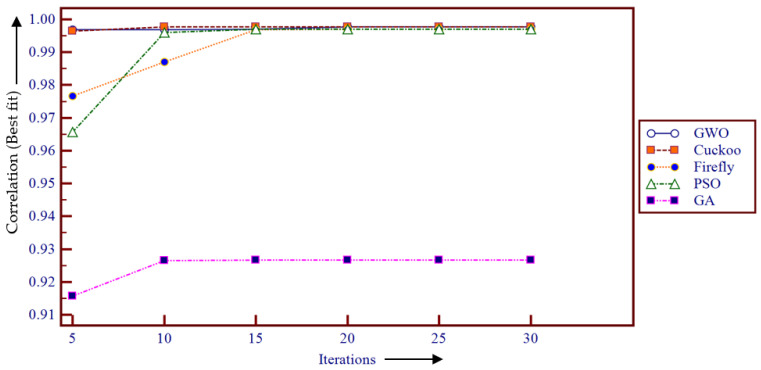
Comparative study of fitness values in meta-heuristic algorithm-based demons registration.

**Figure 10:**
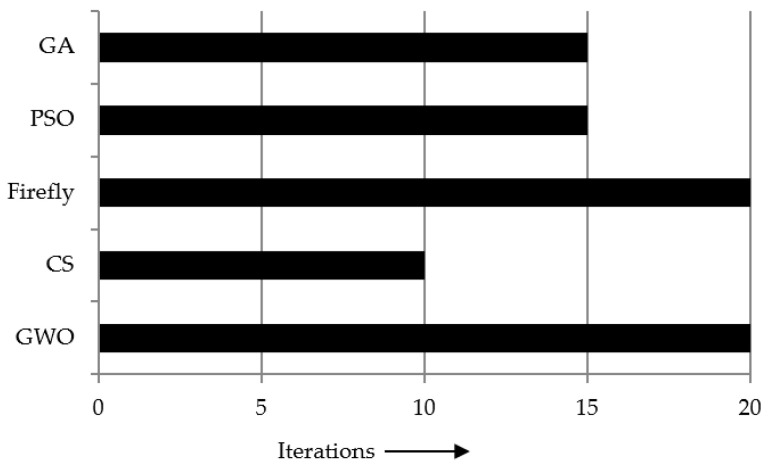
Comparative study of required iterations in meta-heuristic algorithm-based demons registration.

Figure 8 reported that the best/highest fitness (correlation) value is achieved using the PSO-based registration. However, the GWO provided the most stable with less variation in the fitness values. To illustrate the convergence speed, Figure 9 and Figure 10 are plotted to illustrate the comparative study of fitness values in the applied meta-heuristic algorithm-based demons registration using the line plot.

Figure 9 and Figure 10 depicted that the FA took a greater number of iterations for complete convergence, while both the GWO and the GA provided the best fitness value with the least number of iterations (about 5 iterations).

### 6.3. Comparative Study of the GWO-Based Different Demons Registration

The following study is carried out on different demons algorithms in order to establish the superior demons algorithm that worked well with grey-wolf optimization. The first algorithm to undergo the procedure was Wang’s demons. The results are shown in Table 8, which reports the best fitness value was achieved after the 15th iteration.

**Figure 11:**
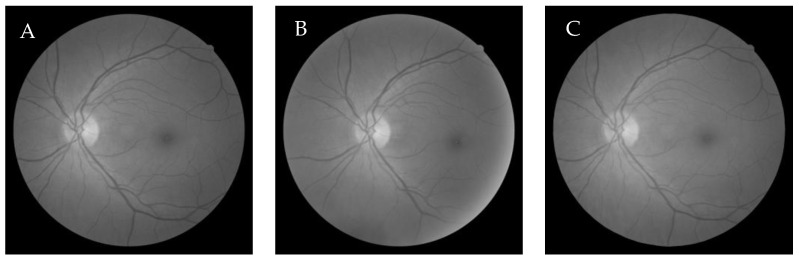
Moving (**A**), fixed (**B**) and registered image (**C**) in grey-wolf optimization-based Wang’s demons registration.

The obtained images are shown in Figure 11. The reference, original, and register image shows the quality of the registered image was quite higher.

The next registration method to undergo the procedure was Tang’s demons. The results are shown in Table 9, which reports the best fitness value was achieved after the 15th iteration. This time the correlation was lower than Wang’s demons.

**Figure 12:**
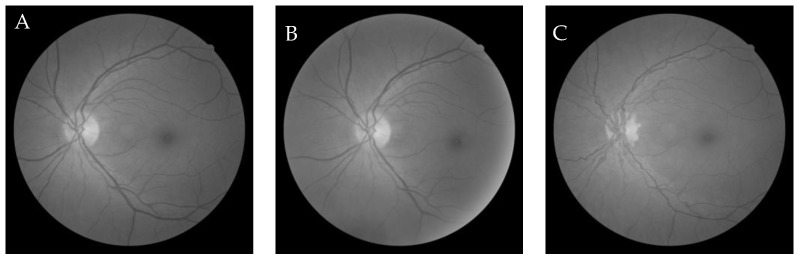
Moving (**A**), fixed (**B**), and registered (**C**) image in grey-wolf optimization-based Tang’s demons registration.

The obtained images are shown in Figure 12. The reference, original, and register image shows the quality of the registered image was quite lower than Wang’s demons.

The final registration method to undergo the procedure was Thirion’s demons. The results are shown in Table 10, which reports the best fitness value was achieved after the 15th iteration. This time also the correlation was lower than Wang’s demons.

**Figure 13:**
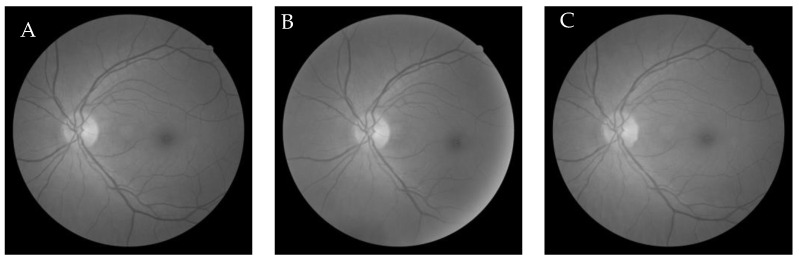
Moving (**A**), fixed (**B**), and registered (**C**) image in grey-wolf optimization-based Thirion’s demons registration.

The obtained images are shown in Figure 13. The reference, original, and register image shows the quality of the registered image was also lower than Wang’s demons.

**Figure 14:**
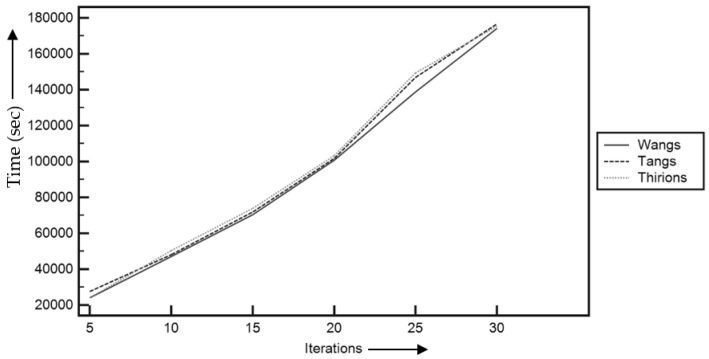
Comparative study of time complexity of different grey-wolf optimization-based demons algorithm.

A comparative analysis of time complexity has been observed in Figure 14, which shows that Wang’s demons took lesser time to find the optimal solution to the registration problem compared to the other two methods.

**Figure 15:**
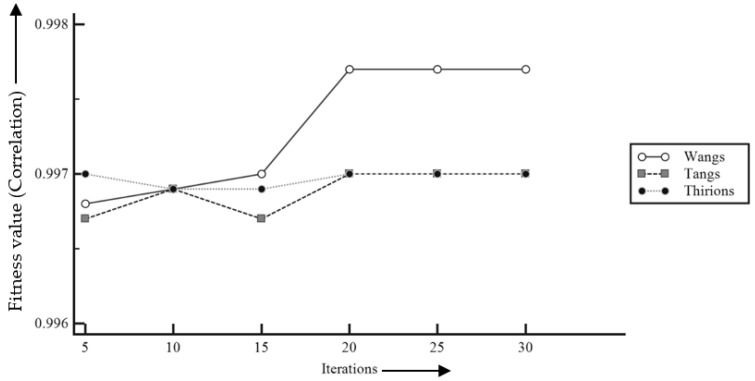
Comparative study of best fitness of different grey-wolf optimization-based demons.

Similarly, a comparative study of best fitness or correlation values has been observed in Figure 15. It shows that Wang’s demons took lesser iterations as well to find the optimal solution to the registration problem compared to the other two methods. The optimal solution itself was better than the other two methods.

Finally, Table 11 reports various image metric’s quantity while exploring grey-wolf optimization-based, different demons algorithms. The mean square error (MSE) refers to the total squared error between the registered and the original image, which is expressed as follows [43]:

(15) $$MSE=\frac{1}{MN}\sum_{y=1}^{M}\sum_{x=1}^{N}{\left[I(x,y)-J(x,y)\right]}^{2}$$

The joint entropy of two images *X* and *Y* is calculated using the following expression [43]:

(16) $$MJE(X,Y)=-\sum_{x\in X}^{}\sum_{y\in Y}^{}P(x,y)\log_{2}[P(x,y)]$$

In addition, the mutual information of the image *X* and *Y* can be defined as [43]:

(17) NMI=entropy(X)+entropy(Y)−MJE(X,Y)

The mean square error, mean joint entropy, and net mutual information values are observed for different demons framework that was based on grey-wolf optimization. Table 11 reports the results of MSE, MJE, NMI, and correlation values. The mean square error was significantly low in Wang’s demons (8.3585 × 10^−5^) registration compared to other two as the higher correlation value (0.9977) of Wang’s supports the claim of obtaining better quality images as a result of grey-wolf optimized Wang’s demons registration, than Tang’s registration and Thirion’s registration’s framework.

**Figure 16:**
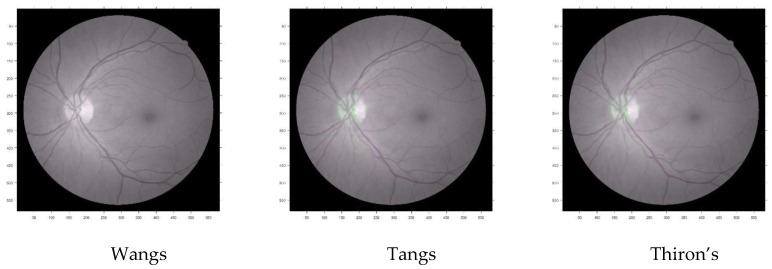
Comparative study of overlapped areas of original and registered images in different grey-wolf optimization-based demons.

Figure 16 showed the overlapped images of the original image and obtained images from three different demons registration frameworks of Wang’s, Tang’s, and Thirion’s demons, based on the grey-wolf optimization.

From the observation in Figure 16 and Table 11, it can be said that grey-wolf optimization-based Wang’s demons performed better in terms of obtaining quality image registration. Although the time complexity study has shown that Thirion’s demons managed to achieve the convergence earlier than Wang’s and Tang’s demons registration based on the GWO algorithm. GWO-based Wang’s demons performed faster during registration and optimization than the other two methods, making it superior among the three demons registration.

## 7. Discussion

Table 12 provides a comparison for 8 of the existing techniques that experimented on retinal image registration algorithms, previously. Among them, in 2005, Chanwimaluang based registration on retinal images obtained from area NIH. They used feature-based [10] image registration and affine registration as the benchmark and reported 95.1% registration accuracy compared to 94.7% and 90.4% accuracy of the other two image registration techniques, respectively. In 2012, Gharabghi proposed retinal image registration using a geometrical [23] feature. In this work, the DRIVE database reported 96% accuracy. Later in 2016, Parekar et al. [62] used feature matching registration on retinal images, which reported a mean error of 1.785, and a standard deviation of 0.974, compared to Generalized Dual-Bootstrap Iterative closest Point’s (GDB-ICP) 3.505 mean error and 2.789 standard deviations. As discussed previously, Hernandez-Matas et al. [22] introduced the rigid registration on retinal images in 2017 using swarm intelligence to solve registration errors generated during the rigid registration process. The results reported a mean error of 0.43, compared to 0.68 of their previous method of Hernandez-Matas et al. 2015 and 7.20 of the RANSAC method. They also reported an improvement of 57.75% on registration accuracy while comparing it with the RANSAC method and 37.93% while comparing it with Hernandez-Matas et al., 2015. Ramli et al. [21] discussed the effect of feature-based registration, in which he used the D-SADDLE feature on retinal image registration. They measured registration accuracy based on the amount of retinal image pair, that was registered successfully. They reported a registration accuracy of 43% but also accepted that the method would fail if the original and reference frame had small overlapping areas. In 2017, Hang et al. proposed a SIFT feature based retinal image registration. In this work, they used bifurcation [63] points as a feature for registering images. They used matching points to measure the accuracy of registration, and the best they could get was 38% accuracy. Like the previous method, their method also failed for registering images having a small overlap area. Li et al. introduced retinal image registration [12], that was feature-based, but the novelty of this method was, it tried to address the image modality issues. They used the Dice coefficient to measure the registration accuracy. In this work, they found the dice coefficient value to be 0.74 ± 0.08 rather than 0.62 ± 0.09 descriptor matching registration and 0.60 ± 0.17 of Miri et al.’s work. Later in 2018, Tang et al. introduced a novel non-rigid [64] point matching registration. They tested the success rate of their method and compared it to UR-SIFT-PIIFD which is a popular approach in multimodel retinal image registration. The success rates are 92.44% and 90.21%, respectively.

The current work discusses the enhancement of the image following the optimization using the grey-wolf algorithm. The proposed work also used other similar demons registration algorithms to compare the current framework’s effectiveness. In addition, the proposed system has been compared with other optimization methods as well, in which it has reported superior results compared to the frameworks based on cuckoo search, firefly algorithm, particle swarm optimization, and genetic algorithm. Finally, in Table 13 the overall improvement as a result of the grey-wolf optimization-based retinal image registration has been reported.

Table 13 clearly shows the improvement of image quality following the image registration using grey-wolf optimized parameters. The increase in correlation between original and registered image (with optimized parameters) clearly indicates the improvement of image quality as an effect of optimization and thus reducing the registration error (by 53.23%). Additionally, the reduction of mean-square error following registration supports the claim. The time complexity has also been addressed during this process as it is visible that a 3.41% reduction of time has taken place as a result of using optimization. The reported case is for 1 frame at a time, hence optimization in image registration will address the time complexity problems of registration frameworks that use medical video of large scale.

## 8. Conclusions

The current work presented an analysis of grey-wolf optimization and its effect on Wang’s demons registration. In the previous work of Lan et al. and Chakraborty et al., Wang’s demons were already proven superior to the existing methods of Tang’s demons and Thirion’s demons. Hence, Wang’s demons was chosen for the current framework. The outcome of the current work showed a grey-wolf optimization-based demons registration framework is one of the fastest and quickest to reach convergence among the existing four meta-heuristic algorithm-based approaches. The results have also indicated that cuckoo search and grey-wolf optimization both produced better resultant images in terms of accuracy of demons registration since it had the highest correlation (0.9977) between original and registered images. The current work is still a pilot in terms of nature. Future work may consist of exploring other meta-heuristic algorithms and combining them with different demons registration framework to analyze the prospect of optimization in the domain of image registration. Future work may include a comparison with the rigid registration method in a similar framework. Multimodal registration is not addressed in the current work, so this might be addressed in future works.

## Figures and Tables

**Table 1:** Default values of the registration parameters in demons registration.

	2-D Filter	Sigma
**Velocity field smoothing kernel**	Gaussian low pass filter	10 pixels
**Alpha (noise) constant**	2.5 dB	

**Table 2:** Grey-wolf optimization algorithm-based Wang’s demons registration.

	5 × 15	10 × 15	15 × 15	20 × 15	25 × 15	30 × 15
Correlation	0.9968	0.9969	0.9970	0.9977	0.9977	0.9977
Time for Optimization	24,153.56	47,175.47	70,458.02	100,752.23	138,726.54	174,113.1
*k*_1_, *k*_2_, *k*_3_	89, 60, 18	97, 79, 18	98, 98, 20	99, 99, 20	99, 99, 20	99, 99, 20

**Table 3:** Firefly algorithm-based Wang’s demons registration.

	5 × 15	10 × 15	15 × 15	20 × 15	25 × 15	30 × 15
Correlation	0.9766	0.9870	0.9969	0.9970	0.9970	0.9970
Time for Optimization	25,104.92	58,984.51	84,369.26	132,714.22	165,263.5	210,714.76
*k*_1_, *k*_2_, *k*_3_	66, 71, 19	87, 50, 18	96, 81, 20	98, 98, 20	98, 98, 20	98, 98, 20

**Table 4:** Cuckoo search algorithm-based Wang’s demons registration.

	5 × 15	10 × 15	15 × 15	20 × 15	25 × 15	30 × 15
Correlation	0.9965	0.9977	0.9977	0.9977	0.9977	0.9977
Time for Optimization	24,820.26	53,485.82	81,148.52	121,591.49	156,132.41	211,056.74
*k*_1_, *k*_2_, *k*_3_	67, 92, 18	99, 99, 20	99, 99, 20	99, 99, 20	99, 99, 20	99, 99, 20

**Table 5:** Genetic algorithm-based Wang’s Demons.

	5 × 15	10 × 15	15 × 15	20 × 15	25 × 15	30 × 15
Correlation	0.9157	0.9265	0.9266	0.9266	0.9266	0.9266
Time for Optimization	4909.18	10,104.6	14,314.98	19,729.19	23,934.43	29,177.21
*k*_1_, *k*_2_,*k*_3_	74, 74, 15	94, 94, 19	96, 96, 19	96, 96, 19	96, 96, 19	96, 96, 19

**Table 6:** Particle swarm optimization algorithm-based Wang’s demons.

	5 × 15	10 × 15	15 × 15	20 × 15	25 × 15	30 × 15
Correlation	0.9657	0.9960	0.9970	0.9970	0.9970	0.9970
Time for Optimization	56,841.91	104,131.62	143,152.31	198,331.62	224,136.44	297,528.52
*k*_1_, *k*_2_,*k*_3_	64, 72, 18	92, 76, 19	98, 98, 20	98, 98, 20	98, 98, 20	98, 98, 20

**Table 7:** The parameters to be optimized.

Total Number of Iterations	120	
**Velocity field smoothing kernel**	**2-D filter**	**Sigma**
	Gaussian low pass filter of window size 60 × 60	10 pixels
**Alpha (noise) constant**	2.5 dB	

**Table 8:** Grey-wolf optimization algorithm-based Wang’s demons registration.

.	5 × 15	10 × 15	15 × 15	20 × 15	25 × 15	30 × 15
Correlation	0.9968	0.9969	0.9970	0.9977	0.9977	0.9977
Time for Optimization	24,153.56	47,175.47	70,458.02	100,752.23	138,726.54	174,113.1
*k*_1_, *k*_2_, *k*_3_	89, 60, 18	97, 79, 18	98, 98, 20	99, 99, 20	99, 99, 20	99, 99, 20

**Table 9:** Grey-wolf optimization algorithm-based Tang’s demons registration.

	5 × 15	10 × 15	15 × 15	20 × 15	25 × 15	30 × 15
Correlation	0.9967	0.9969	0.9967	0.9970	0.9970	0.9970
Time for Optimization	27,703.23	48,149.94	71,915.53	101,793.91	146,753.1	175,665.3
*k*_1_, *k*_2_, *k*_3_	74, 98, 18	79, 99, 20	80, 91, 18	98, 98, 20	98, 98, 20	98, 98, 20

**Table 10:** Grey-wolf optimization algorithm-based Thirion’s demons registration.

	5 × 15	10 × 15	15 × 15	20 × 15	25 × 15	30 × 15
Correlation	0.9970	0.9969	0.9969	0.9970	0.9970	0.9970
Time for Optimization	23,927.88	50,362.82	73,978.46	103,359.34	149,332.8	175,360.6
*k*_1_, *k*_2_, *k*_3_	96, 94, 19	53, 99, 19	68, 98, 20	98, 98, 20	98, 98, 20	98, 98, 20

**Table 11:** Study of grey-wolf optimization algorithm-based demons registration.

Optimized Values	Wang’s Demons	Tang’s Demons	Thirion’s Demons
**MSE**	8.3585 × 10^−5^	2.7314 × 10^−4^	2.6885 × 10^−4^
**MJE**	5.3007	5.3464	5.3456
**NMI**	0.4920	0.4493	0.4500
**Correlation**	0.9977	0.9970	0.9970

**Table 12:** Summary of studies that presented various types of retinal image registration.

Sl. No.	Year	Technique Used	Database Used	Size of Data	Performance Measures	Limitations
1	Gharabaghi 2012 [23]	Geometrical feature-based registration	DRIVE	20	Accuracy of 96%	Multimodal registration not taken into account
2	Chanwimaluang 2005 [10]	Area-based registration and Feature-based registration, Affine registration	NIH	1008	Accuracy of 95.1%	The time complexity of processing not discussed/addressed
3	Tang et al. 2018 [64]	Non-rigid point matching registration	http://imagebank.asrs.org/	200	Accuracy of 92.44%	The feature matching technique wasn’t robust. Time complexity not discussed
4	Hernandez-Matas et al. 2017 [22]	Rigid registration with PSO	FIRE	116	Highest accuracy of 71.11% was observed while applying on different methods	A comparative study of other optimization method was missing. Discussion about the inclusion of non-rigid registration was missing.
5	Ramli et al. 2017 [21]	Feature-based Registration (D-Saddle feature)	FIRE	134	Registration accuracy 43%	Applied on Mono-modal images only. The method fails for small overlapping areas. Time complexity not addressed
6	Hang et al. 2017 [63]	SIFT Feature based registration	FIRE	134	2.52 seconds/frame	The method fails when overlapping areas of the two images are very small.
7	Li et al. 2018 [12]	Feature-based multimodal registration	University Eye Clinic Maastricht, Maastricht	600	Dice coefficient was measured (Average failure of 10.37)	Comparative analysis was weak. Time complexity not discussed
8	Parekar et al. 2016 [62]	Feature matching registration	DRIVE	12	Mean error (2.789) and std dev (0.974)	The concept was based on similarity transformation

**Table 13:** Effect of grey-wolf optimization-based demons registration.

	Original Parameters	Optimized Parameters	Improvements
**Correlation**	0.4666	0.9977	53.2324346 (Increase)
**MSE**	3.6971 × 10^−4^	8.3585 × 10^−5^	77.39173947 (Decrease)
**MJE**	5.3615	5.3007	1.134011004 (Decrease)
**NMI**	0.4408	0.4920	10.40650407 (Increase)
**Registration time/Frame (sec)**	443.242591	428.124839	3.41071736 (Decrease)
